# Reorganization and plastic changes of the human brain associated with skill learning and expertise

**DOI:** 10.3389/fnhum.2014.00035

**Published:** 2014-02-04

**Authors:** Yongmin Chang

**Affiliations:** Department of Molecular Medicine and BK21 Plus KNU Biomedical Convergence Program, Kyungpook National University School of MedicineDaegu, South Korea

**Keywords:** expertise, plasticity, reorganization, neuro imaging, skill learning

## Abstract

Novel experience and learning new skills are known as modulators of brain function. Advances in non-invasive brain imaging have provided new insight into structural and functional reorganization associated with skill learning and expertise. Especially, significant imaging evidences come from the domains of sports and music. Data from *in vivo* imaging studies in sports and music have provided vital information on plausible neural substrates contributing to brain reorganization underlying skill acquisition in humans. This mini review will attempt to take a narrow snapshot of imaging findings demonstrating functional and structural plasticity that mediate skill learning and expertise while identifying converging areas of interest and possible avenues for future research.

## Introduction

Neuroplasticity, which refers to the brain's ability to change its structure and function, is not an occasional state of the brain, but rather the normal ongoing state of the human brain throughout the life span (Zilles, 1992; Pascual-Leone et al., 2005; Kempermann, 2006; Jancke, 2009). Plastic changes in the human brain lead to brain reorganization that might be demonstrable at the level of behavior, anatomy, and function and down to the cellular and even molecular levels (Kolb and Whishaw, 1998; Kelly and Garavan, 2005; Kleim et al., 2006).

Intentional practice in sports and music has been shown to contribute to acquisition of expertise (Schlaug, 2001; Baker et al., 2003; Hutchinson et al., 2003; Lotze et al., 2003; Calvo-Merino et al., 2005; Ericsson, 2005; Cross et al., 2006; Hung et al., 2007; Nielsen and Cohen, 2008). Acquisition of expertise is accompanied by structural and functional changes of the brain and the advent of brain imaging methods has bolstered the study of these changes in the human brain. Understanding of the neural mechanisms underpinning expertise may provide a basis for determining what types of practice or training are most likely to be beneficial for performance enhancement. This knowledge may also provide a clue as to why some people show improvement at different rates than others or reach much higher levels of achievement. Thus, the study of plastic changes associated with skill learning and expertise in the human brain is one of the most challenging areas of current neuroscience research.

This mini review provides a summary of the *in vivo* imaging evidence of longitudinal and cross-sectional studies on structural and functional plasticity of the human brain in skill learning and expertise with emphasis on sports and music. In the literature, a cross-sectional approach has been most widely used, and many interesting findings have been reported. However, one of the criticisms of cross-sectional studies is that the differences in brain organization are possibly correlational, and, thus, caution should be used in order not to draw overly strong causal inferences from the cross-sectional data. The concept of plasticity can involve many levels of organization involving molecular, neuronal, or chemical events, and these molecular views of neuroplasticity are beyond the scope of this mini review.

## Structural neuroplasticity in skill learning and expertise

### Cross-sectional studies

Cross-sectional imaging studies have demonstrated structural changes of the human brain as a result of experience and learning in sports and music (Amunts et al., 1997; Gaser and Schlaug, 2003; Bangert and Schlaug, 2006; Jacini et al., 2009; Jäncke et al., 2009; Park et al., 2009; Hänggi et al., 2010; Wan and Schlaug, 2010; Wei et al., 2011; Di Paola et al., 2013). For example, Jacini et al. (2009) reported that elite judo players had significantly higher gray matter volume in the frontal lobe, related to motor planning and execution and in regions of the prefrontal cortex, related to working memory and cognitive processes, compared to control subjects. Training induced enlargement in gray matter structure was not limited to brain regions associated with motor planning and execution. When compared to age-matched control subjects, world-class mountain climbers showed significantly larger vermian lobule volumes, possibly associated with highly dexterous hand movements and eye-hand coordination in detection of and correction of visuomotor errors (Di Paola et al., 2013). In the music domain, with measurement of the length of the posterior wall of the precentral gyrus as an estimate of the size of the hand motor area, Amunts et al. (1997) identified substantial structural differences in the hand motor area between professional musicians and non-musicians: in general, the hand motor area was larger in professional musicians than in non-musicians. More importantly, the authors also found that the measures of hand motor area on both hemispheres showed correlation with the age of commencement of musical training, implying that earlier musical training results in a stronger impact on structural changes in the hand motor area.

In a study using the voxel-based morphometry (VBM) technique, it was found that skilled golfers (professional and low handicap golfers) had larger gray matter volumes in a fronto-parietal network, including premotor and parietal areas (Jäncke et al., 2009). Using the VBM approach, Gaser and Schlaug (2003) reported that professional keyboard players showed differences in gray matter volume in motor, auditory, and visual-spatial brain regions when compared with a matched group of amateur players and non-keyboard players. While the majority of studies on structural neuroplasticity have reported increased gray matter density or volume in expert brains, few studies have reported on the inverse relationship, that is, decreased gray matter volume (Draganski et al., 2006; Hänggi et al., 2010). The several possible reasons for discrepant findings were suggested (Hänggi et al., 2010).

A handful of studies have investigated differences in white matter structure between experts and non-experts, using diffusion tensor imaging (DTI); however, the results have been inconsistent. Using DTI, Jäncke et al. (2009) demonstrated decreased white matter volume and fractional anisotropy (FA) values in several brain structures, including the corticospinal tract (CST), in skilled golfers, compared with less-skilled golfers. Additional evidence for decreased white matter volume and FA values was reported in a study of professional ballet dancers (Hänggi et al., 2010). Contrary to decreased FA values in white matter structures, a very recent study on professional gymnasts showed increased FA values in the bilateral CST in elite gymnasts, possibly in response to long-term gymnastic training as compared to the control subjects (Wang et al., 2013). Inconsistent results have also been reported in the music domain. Imfeld et al. (2009) reported significantly lower FA values in both the left and the right CST in professional musicians compared to non-musicians. However, in another study, pianists who practiced frequently showed higher FA values (Han et al., 2009). Therefore, it appears that acquisition of further evidence will be necessary in order to make a conclusion with regard to whether specific structural changes in white matter can be induced by extensive training.

### Longitudinal studies

To date, only a small number of longitudinal studies have investigated structural brain reorganization as a result of experience and learning. Draganski et al. (2004) investigated the training effect of juggling in inexperienced young jugglers. After a 3-month training period, subjects in the training group showed changes in gray matter density in the intraparietal sulcus and the midtemporal area of visual cortex. The intraparietal sulcus is involved in transforming retinotopic into body centered information necessary to visually control movements. The midtemporal area of visual cortex is a highly specialized brain area for analyzing visual movement information. Of particular interest, the authors also found that after another 3 months without juggling practice, the increase in gray matter density following practice had diminished in all subjects in juggling practice, indicating that structural plasticity is reversible. In a recent study of 60-year old elderly individuals who were able to learn juggling, gray matter changes related to skill acquisition were observed in the midtemporal area of visual cortex similar to that found in young subjects, suggesting that age is not in itself a limiting factor for structural brain plasticity driven by skill learning (Boyke et al., 2008). In a more recent longitudinal study using VBM, in golf novices between the ages of 40 and 60 years, 40 h of golf training showed an association with gray matter increases in a task-relevant cortical network encompassing sensorimotor regions and areas belonging to the dorsal stream (Bezzola et al., 2011). More importantly, in that study, a strong positive relationship was observed between the increase in gray matter and training intensity in the parieto-occipital junction (POJ), a critical structure of the dorsal stream. A recent review provided evidence of a close association of the POJ with visuomotor processes, particularly in the on-line control and on-line correction of visually guided arm movements (Kravitz et al., 2011). For musical training, Hyde et al. (2009) found that 6-year-old children receiving instrumental musical training for 15 months showed structural change in brain areas such as the precentral gyrus, which is known to be involved in control of playing a musical instrument. Most of these brain areas are part of the cortical motor system; however, structural changes in the auditory system, such as the Heschl gyrus and the corpus callosum, were also observed. These structural changes in the brain showed correlation with performance on various auditory and motor tasks. In addition, in the music domain, the evidence suggests that training-induced plasticity in musicians appears to be most prominent in those who engaged in practice early in childhood (for a review, see Wan and Schlaug, 2010).

## Functional neuroplasticity in skill learning and expertise

### Cross-sectional studies

In motor function, a common finding is the functional enlargement or focused activation of the motor area involved in control of that particular skill (Krings et al., 2000; Pearce et al., 2000; Lotze et al., 2003; Haslinger et al., 2004; Meister et al., 2005; Bangert and Schlaug, 2006). For example, Pearce et al. (2000) reported that the cortical representation of the hand used for playing is larger in professional racquet ball players as compared with novices. In music, one study demonstrated a differential brain adaptation depending on instrument played (Bangert and Schlaug, 2006). More specifically, keyboard players had the left motor area more pronounced as they predominantly use the right hand. In contrast, string players had the right motor area pronounced as the left hand is crucially engaged while playing.

Recent neuroimaging studies have attempted to elucidate the neural activity during action observation in expert brain (Calvo-Merino et al., 2006; Pilgramm et al., 2010; Kim et al., 2011). For example, Calvo-Merino et al. (2006) demonstrated the neural bases of motor influences on action observation in expert ballet dancers. They have shown an effect of motor expertise on neural activation within the ventral premotor area and also stronger activation in the inferior parietal and cerebellar regions when observing dance videos, suggesting that the action observation network is more extended than previously suggested (Di Pellegrino et al., 1992). For motor planning in expertise, an fMRI study using motor imagery task, which refers to the mental rehearsal of motor acts, demonstrated that the task-related neural networks of expert golfers are focused and efficiently organized, whereas novices have difficulty filtering out irrelevant information (Milton et al., 2007). This finding is consistent with the notion of relative economy (neural efficiency) in the cortical processes of elite athletes during the specific challenge in which they are highly practiced. Similar finding was also observed in professional musicians. Lotze et al. (2003) reported that professional violinists showed focused cerebral activations in the contralateral primary sensorimotor cortex, the bilateral superior parietal lobes, and the ipsilateral anterior cerebellar hemisphere as compared to amateur violinists during the imagination of violin-playing movements.

As for the visuospatial abilities in sport, evidences seem to suggest that experts differ in visuospatial abilities directly tied to their domain of expertise. For example, one study reported that expert athletes did not differ in their visuospatial capacity than novices as measured on the general visuospatial test (Furley and Memmert, 2010). However, a recent study using fMRI reported quantitative differences in brain activation during visuospatial processing between elite rugby players and novices, indicating the possible existence of a strategy (a bird's eye view) regarding visuospatial cognitive processing for elite rugby players that differs from that of novices (Sekiguchi et al., 2011). More recently, Seo et al. (2012) investigated possible difference in cognitive strategy between archery experts and novices in visuospatial working memory processing. According to their results, archery experts have increased activation in cortical regions important for visuospatial attention and working memory, suggesting that degree of expertise may modulate higher order brain functioning. Taken together, these studies therefore demonstrated that the differences in visuospatial abilities are pronounced in specific domain but those differences did not transfer outside the domain to general visuospatial ability. The possible modulation on function of working memory and attention by expertise was also recently demonstrated in music training. In their multilevel cross-sectional study, Oechslin et al. (2013) found evidence for stepwise modulation of brain responses according to level of music expertise in a fronto-temporal network hosting functions of working memory and attention.

### Longitudinal studies

For motor skill acquisition, previous studies using fMRI demonstrated that learning of sequential finger movements initially leads to a functional expansion in the primary motor cortex (M1) and this change in M1 follows more dynamic, rapid changes in the cerebellum, striatum, and other motor-related cortical areas, suggesting an experience-dependent shift of activation from a cerebellar–cortical to a striatal–cortical network with extended practice (Karni et al., 1995; Doyon et al., 2002). In addition, repetition of movements has been suggested to result in motor memories in the primary motor cortex and probably other cortical areas that encode the kinematic details of the practiced movements (Classen et al., 1998; Butefisch et al., 2000; Stefan et al., 2005; Cross et al., 2009). Of particular interest, previous studies have demonstrated that motor memory can also be encoded by action observation and this form of action observation can enhance the effects of motor training on memory encoding, possibly through modulation of intracortical excitatory mechanisms (Stefan et al., 2005; Celnik et al., 2006).

Formation of multisensory connection during motor learning has often been reported in music. In a longitudinal EEG study (Bangert and Altenmüller, 2003), beginning pianists, who had never played an instrument before, were trained on a computer piano over a period of 5 weeks. They listened to short piano melodies, and, after a brief pause, they were then required to replay the melodies using their right hand. After 5 weeks of practice, listening to piano tunes produced additional activity in the sensorimotor regions and in turn, playing on a keyboard produced additional activity in the auditory regions. Therefore, this study nicely demonstrates how dynamic brain adaptations accompany these multisensorimotor learning processes. In another longitudinal study using fMRI (Herdener et al., 2010), the neural responses of musical students in acoustic novelty detection were compared before and after two semesters of intensive aural skills training. Following the training period, hippocampal responses to temporal novelty in sounds were increased in music students. A previous study suggested involvement of the hippocampus in various forms of novelty detection in addition to its role in memory (Knight, 1996; Strange et al., 1999). Therefore, this study provides evidence for functional plasticity in the adult hippocampus related to musical training.

## Concluding remarks

**Figure 1:**
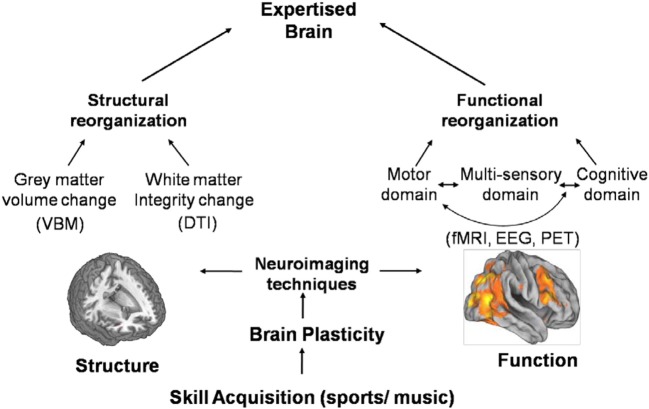
**Advances in neuro imaging technique have provided new insights into the neuroplastic changes underlying skill learning and expertise at both structural and functional levels**. At structural level, a main finding is increased gray matter volume or density of brain areas associated with skill learning. In functional reorganization, functional imaging evidence has shown that functional neuroplasticity occurs not only in the motor domain but also in cognitive and perceptual domains associated with improved performances.

Over the past decades, advances in human brain imaging have provided new insights into the neuroplastic changes underlying skill learning and expertise in both sports and music (Table 1). These plastic changes can be seen at both structural and functional levels (Figure 1). A main finding of structural plasticity is increased volume and gray matter density of brain areas involved in control of the practiced task. Another major finding in structural plasticity is that experience dependent structural changes can disappear when practicing stops, indicating that structural plasticity is possible in all directions. In musical expertise, one of the distinctive features of structural neuroplasticity is that brain plasticity can be found more clearly if practice starts at a young age. That is, a period might exist, beyond which music-induced structural changes and learning effects are less pronounced. Unfortunately, such studies on a sensitive period are missing in the sport domain.

**Table 1:** **Imaging evidences for structural and functional plasticity in sports and music**.

**Study**	**Skill**	**Design**	**Method**	**Main findings**
**STRUCTURAL PLASTICITY**				
Jacini et al., 2009	Sports	Cross-sectional	VBM	Judo players showed larger GM volume in frontal and prefrontal cortex
Jäncke et al., 2009	Sports	Cross-sectional	VBM, DTI	Golfers showed Larger GM volumes in premotor and parietal cortices; smaller FA along the internal and external capsule and the parietal operculum
Di Paola et al., 2013	Sports	Cross-sectional	VBM	Mountain climbers showed significantly larger vermian lobule volumes
Draganski et al., 2004	Sports	Longitudinal	VBM	Three months' practice-induced GM expansion in mid-temporal area and posterior intraparietal sulcus, followed by a decreased to baseline levels after 3 months with no practice
Bezzola et al., 2011	Sports	Longitudinal	VBM	Forty hours of golf training showed an association with gray matter increases in a task-relevant cortical network
Amunts et al., 1997	Music	Cross-sectional	MRI	Hand motor area was larger in professional musicians than in non-musicians
Gaser and Schlaug, 2003	Music	Cross-sectional	VBM	GM volume differences in sensorimotor cortex, premotor cortex, and cerebellum
Han et al., 2009	Music	Cross-sectional	VBM, DTI	Musician showed higher GM density in sensorimotor cortex and cerebellum; higher FA in internal capsule
Hyde et al., 2009	Music	Longitudinal	DBM	Fifteen months of musical training in early childhood showed structural change in brain areas which are known to be involved in control of playing a musical instrument
**FUNCTIONAL PLASTICITY**				
Pearce et al., 2000	Sports	Cross-sectional	TMS	Cortical representation of the hand used for playing is larger in professional racquet ball players
Milton et al., 2007	Sports	Cross-sectional	fMRI	Elite athletes showed neural efficiency in the cortical processes during the specific challenge in which they are highly practiced
Sekiguchi et al., 2011	Sports	Cross-sectional	fMRI	Elite rugby players differ in visuospatial abilities directly tied to their domain of expertise
Doyon et al., 2002	Sports	Longitudinal	fMRI	Shift of activation from the cerebellar cortex to the dentate nucleus during early learning, and from a cerebellar–cortical to a striatal–cortical network with extended practice
Cross et al., 2009	Sports	Longitudinal	fMRI	Emergence of action resonance processes in the human brain based on 5 day observational learning of dance sequence without physical practice
Lotze et al., 2003	Music	Cross-sectional	EMG	Professional violinists showed focused cerebral activations in the motor network as compared to amateur violinists during the imagination of violin-playing movements
Oechslin et al., 2013	Music	Cross-sectional	fMRI	Levels of musical expertise stepwise modulate higher order brain functioning
Bangert and Altenmüller, 2003	Music	Longitudinal	EEG	Auditory-sensorimotor co-activity occurred within only 20 min and the effect was enhanced after 5-week training, contributing elements of both perception and action to the mental representation of the instrument
Herdener et al., 2010	Music	Longitudinal	fMRI	Following the aural skills training, hippocampal responses to temporal novelty in sounds were increased

*MRI, magnetic resonance imaging; VBM, voxel-based morphometry; GM, gray matter; DTI, diffusion tensor imaging; FA, fractional anisotropy; DBM, Deformation based morphometry; TMS, transcranial magnetic stimulation; fMRI, functional MRI; EMG, Electromyography; EEG, electroencephalography*.

In functional reorganization, a common finding is the functional enlargement or focused activation of the motor area involved in control of that particular skill. In addition, because expert performance is mediated by cognitive and perceptual motor skills, functional imaging evidence has shown that functional neuroplasticity occurs not only in the motor domain but also in cognitive and perceptual domains associated with improved performances. Furthermore, in music, evidence has demonstrated a strong coupling of sensorimotor and auditory processing for music expertise. Practice in playing a music instrument involves constant improvement of complex sensory-motor coordination through repeated execution of motor activities under the controlled monitoring of the auditory system.

Despite accumulation of significant imaging evidence, as discussed in the current mini-review, understanding of mechanisms underlying these plastic changes is still far from complete—which opens a broad avenue for future research. For example, neuroplasticity can be traced to cellular and molecular levels, and, thus, one of the main challenges is linking human brain imaging findings to the underlying molecular events. Because the poor specificity of macroscopic MR imaging signals largely precludes molecular information, other non-invasive approaches would be needed. Of these methods, molecular imaging using positron emission tomography (PET) is a good candidate. Although there is still a lack of prospective studies on plasticity, integration of PET into MRI with simultaneous recordings of molecular and hemodynamic brain responses opens new and promising prospects for the future (Judenhofer et al., 2008). Another challenge for the understanding of neural mechanisms underlying plastic changes is time scale of neural activity, because the temporal resolution of fMRI in the order of seconds is approximately three orders of magnitude away from the time scale of neural events in milliseconds order. Therefore, for measurement of brain activity on a time scale of neuronal activity and for assessment of specific neurophysiological events in human, combined fMRI with non-invasive electrophysiological methods such as electroencephalography (EEG) would be beneficial for simultaneous measurement of neuronal and neural brain responses. Combined EEG and fMRI studies can thus take advantage of both, the good spatial resolution of fMRI and the good temporal resolution of EEG (Thees et al., 2002; Debener et al., 2005).

### Conflict of interest statement

The author declares that the research was conducted in the absence of any commercial or financial relationships that could be construed as a potential conflict of interest.
